# Calcium-stores mediate adaptation in axon terminals of Olfactory Receptor Neurons in Drosophila

**DOI:** 10.1186/1471-2202-12-105

**Published:** 2011-10-24

**Authors:** Meena S Murmu, Jacques Stinnakre, Eléonore Réal, Jean-René Martin

**Affiliations:** 1 Imagerie Cérébrale Fonctionnelle et Comportements, Neurobiologie et Développement (N&D), CNRS, UPR-3294, 1 Avenue de la Terrasse, Bâtiment 32/33, 91198, Gif-sur-Yvette, Cedex, France

## Abstract

**Background:**

In vertebrates and invertebrates, sensory neurons adapt to variable ambient conditions, such as the duration or repetition of a stimulus, a physiological mechanism considered as a simple form of non-associative learning and neuronal plasticity. Although various signaling pathways, as cAMP, cGMP, and the inositol 1,4,5-triphosphate receptor (InsP_3_R) play a role in adaptation, their precise mechanisms of action at the cellular level remain incompletely understood. Recently, in *Drosophila*, we reported that odor-induced Ca^2+^-response in axon terminals of olfactory receptor neurons (ORNs) is related to odor duration. In particular, a relatively long odor stimulus (such as 5 s) triggers the induction of a second component involving intracellular Ca^2+^-stores.

**Results:**

We used a recently developed *in-vivo *bioluminescence imaging approach to quantify the odor-induced Ca^2+^-activity in the axon terminals of ORNs. Using either a genetic approach to target specific RNAs, or a pharmacological approach, we show that the second component, relying on the intracellular Ca^2+^-stores, is responsible for the adaptation to repetitive stimuli. In the antennal lobes (a region analogous to the vertebrate olfactory bulb) ORNs make synaptic contacts with second-order neurons, the projection neurons (PNs). These synapses are modulated by GABA, through either GABAergic local interneurons (LNs) and/or some GABAergic PNs. Application of GABAergic receptor antagonists, both GABA_A _or GABA_B_, abolishes the adaptation, while RNAi targeting the GABAB_R _(a metabotropic receptor) within the ORNs, blocks the Ca^2+^-store dependent component, and consequently disrupts the adaptation. These results indicate that GABA exerts a feedback control. Finally, at the behavioral level, using an olfactory test, genetically impairing the GABA_B_R or its signaling pathway specifically in the ORNs disrupts olfactory adapted behavior.

**Conclusion:**

Taken together, our results indicate that a relatively long lasting form of adaptation occurs within the axon terminals of the ORNs in the antennal lobes, which depends on intracellular Ca^2+^-stores, attributable to a positive feedback through the GABAergic synapses.

## Background

Adaptation, a reduction of the response to repeated stimuli, is considered to be a simple form of non-associative learning, as well as one of the most simple and widespread forms of neuronal plasticity. Functionally, adaptation extends the operating range of sensory systems over a large range of stimulus intensities [1]. Sensory systems are modified by experience through multiple mechanisms operating in a large variable time scale, ranging from milliseconds, seconds, minutes or even weeks, suggesting different temporal mechanisms of adaptation. Vertebrate ORNs, like other types of sensory neurons, adapt to a given stimulus, by time-dependent modification in sensitivity. Indeed, odor response declines during prolonged odor stimulation [2,3]. In mice, exposure to an odorant over a period of weeks results in increased odorant sensitivity [4], while in humans, psychophysical studies have revealed that the perceived intensity of an odorant continuously decreases for minutes after odorant exposure [5]. In invertebrates such as *C. elegans*, prolonged exposure to an odorant yields a diminished response to the odorant for several hours [6]. Thus, the kinetics of the changes in adaptation depends upon the stimulus context, as well as its duration and/or its frequency of repetition.

Olfactory stimuli generate cellular responses by modifying the levels of different second messengers in both vertebrates and invertebrates. Cyclic adenosine monophosphate (cAMP) [7,8], cGMP [9] and the inositol 1,4,5-trisphosphate (InsP_3_) signaling pathways [10] have been implicated, suggesting that olfactory transduction may require parallel or interacting pathways [11,12]. However, the precise subcellular localization of each secondary messenger, for example, whether they are present in the dendrites, cell bodies or axon terminals, as well as their precise kinetics and interactions, remain largely unknown. For example, the primary response of ORNs to odor-ligands is a rapid rise in cAMP, which directly opens Ca^2+^-permeable cyclic nucleotide-gated (CNG) ion channels [12]. Thus, cAMP, CNG-dependent ion channels, and Ca^2+^-dependent mechanisms have been proposed to mediate adaptation [8]. However, several Ca^2+^-independent mechanisms have also been implicated in adaptation, including odorant receptor phosphorylation by protein kinase A [13] and G-protein coupled receptor kinase 3 (GRK3) [14]. cGMP is also a likely part of the apparatus mediating adaptation, since a particular form of adaptation operating on a time scale of minutes, termed long-lasting adaptation (LLA), has been described, which is dependent on cGMP through carbon monoxide (CO) [9,15]. Thus, based on the time scale of their kinetics, there is evidence for the coexistence of at least three different types of odor adaptation in a single ORN: short-term adaptation, desensitization and long-lasting adaptation [12].

In *Drosophila*, a mutation in the inositol 1,4,5-trisphosphate receptor (InsP_3_R) affects olfactory adaptation [16], when measured either behaviorally or physiologically (such as an electroantennogram). However, as these studies were based on mutations, which affect all cells of the organism that express the InsP_3_R gene, the precise mechanisms at the cellular and molecular levels, and the neurons in which adaptation occurs and particularly in the ORNs, still remains to be elucidated. Recently, taking advantage of a new *in-vivo *bioluminescence imaging technique [17], allowing continuous monitoring of neuronal Ca^2+^-activity over a long time range, we have shown that the odor-induced Ca^2+^-response in the axon terminal of ORNs is related to odor duration [18]. A short odor-stimulus (< 1s) induces a short Ca^2+^-response due to the opening of the Voltage-Gated-Calcium-Channel (VGCC), while a long odor stimulus, such as 5s, generates through the InsP_3_R and/or the ryanodine (RyR) receptors in addition a slightly delayed second component, which relies on intracellular Ca^2+^-stores. Here, advancing beyond these findings, using the binary P[GAL4] expression system to specifically target in the ORNs, with the P[Or83b-GAL4]) line [19] different RNAi constructs concomitantly with the GFP-aequorin (GA) bioluminescent probe, complemented by a pharmacological approach, we describe a long-lasting form of adaptation. We also bring new insights into the activation of the InsP_3_R and RyR signaling pathways, through feedback control involving GABA in the antennal lobes.

## Results

**Figure 1:**
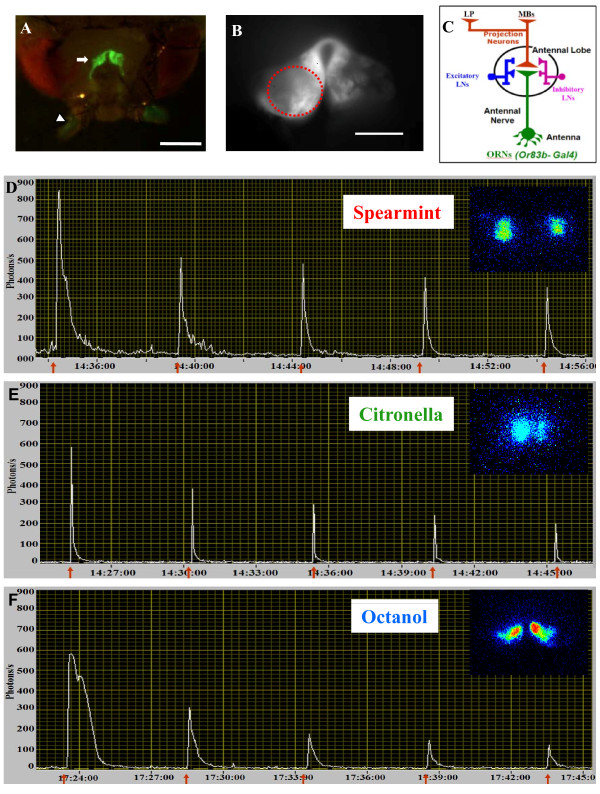
**Odor-induced Ca^2+^-responses in axon terminals of ORNs of a control fly (Or83b,GA/CS)**. (A) Combined dim-light and fluorescence images showing the ORNs in the antennae (arrowhead) and their synaptic terminals (arrow) in the antennal lobes (Leica MZ FLIII binocular, Scale bar = 100 μm). (B) Fluorescence image of the antennal lobes taken at the beginning of the experiment and used as reference image. The red-dashed circle represents the ROI (Region of Interest) from which the light emission is quantified (Scale bar = 50 μm). (C) Schematic drawing of the local neuronal network in the antennal lobe. Ca^2+ ^-activity is recorded in the axon terminals of the ORNs (in green), LNs: local interneurons, LP: lateral protocerebrum, MBs: Mushroom-bodies. (D,E,F) A representative bioluminescent Ca^2+ ^-activity profile evoked by 5 applications of 5 s odor duration (red arrow) at 5 min-intervals of spearmint (D), citronella (E), octanol (F). Inset: a bioluminescence image (accumulation time: 10 s) of the first odor application for each odor. We remark that the activated region is different for each odor (odor specific). ORNs response adapts to repeated odor stimulation (see also movie in Additional file 1).

**Figure 2:**
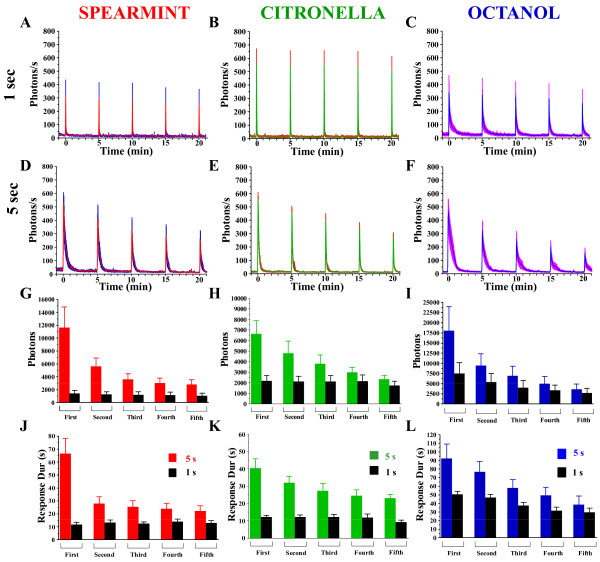
**ORNs adapt to long-lasting and repeated odor stimulation**. (A-F) Mean (+/- SEM) of amplitude of the response (photons/s) of different flies, versus time, of the Ca^2+ ^-induced response (within the ROI) evoked by 1 s (A,B,C) or 5 s (D,E,F) application of spearmint (red-left column), citronella (green-middle column), or octanol (blue-right column) (Spearmint: red line = mean, blue = SEM) (Citronella: green line = mean, and red = SEM) (Octanol: blue line = mean, red = SEM) (same color-code for Figures 4 to 8). (G-I) Mean of the total number of photons of the 5 successive applications, for each odor. (J-L) Mean of the duration of the response (s). n = 5-9 flies for each condition. Values are means +/- SEM. Statistics: for the Amplitude (A-F): One-Way ANOVA, for the total photons and duration (G-L): Two-Way ANOVA (see Table 1 for complete statistical values). Remark that the ordinates (y-axis) of figures (G-I) and (J-L) have a different scale (also for other figures).

GFP-aequorin (GA) is a Ca^2+^-sensitive bioluminescent photoprotein having an excellent signal-to-noise ratio that can be utilized for continuous long-term imaging [17,20,21]. We have developed an *in-vivo *preparation that allows the detection of odor-dependent changes in bioluminescence intensity in the sensory neurons of a living fly (Figure 1A,B,C). Using the fluorescent property of the GFP moiety of the chimeric GA protein, targeted expression of GA (Or83b,UAS-GA/CS) reveals the anatomical organization of ORNs. Delivery of an odor, spearmint (sp), citronella (ci), or octanol (oct), during 5 s to the antennae induces a significant increase in the bioluminescence intensity at ORN synaptic terminals in the antennal lobe, reflecting activity-dependent increases in cytosolic Ca^2+^-concentration (Figure 1D,E,F). The light-induced responses are odor-specific, considering their intensity, duration, shape as well as the activated region in the olfactory lobe (Figure 1D,E,F, Figure 2, and the movie in Additional file 1).

### Ca^2+^-response correlates with odor-duration and repetition frequency of the stimulus

**Figure 3:**
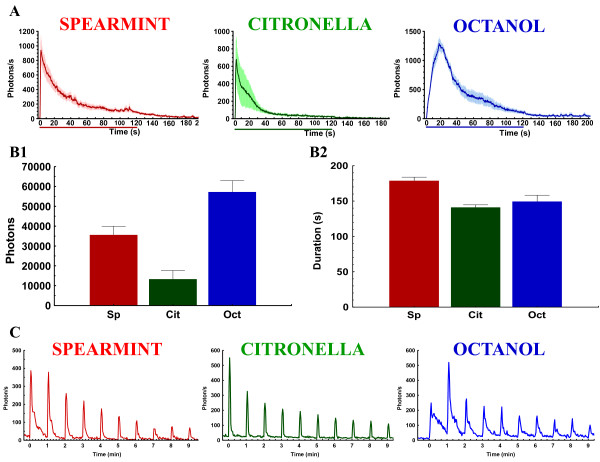
**Odor-induced Ca^2+^-activity is maintained all along the 2-min odor application, and is sensitive to the frequency of the odor-repetition**. (A) Mean of the amplitude (+/-SEM) of the response (photons/s) versus time, of the Ca^2+^-induced response evoked by a long odor application (2 min) (represented by the colored bar below the abscissa) for the three tested odors (n = 6 flies for each odor). Interestingly, we note that the Ca^2+^-response in the axon terminal is maintained (although it decreases) at least during the 2-min odor application (for each of the three tested odors), but rapidly decreases when the odor application is stopped. (B) Total amount of emitted photons and duration of the response of the 2-min odor application for each odor. The duration of the response is longer than 2 min (Sp = 178 s, Ci = 141 s, Oct = 149 s). C) Amplitude of the response (photons/s) of a representative fly, versus time, of the Ca^2+^-induced response (within the ROI) evoked by 5 s application of spearmint, citronella, and octanol, repeated 10 times at 1 min-interval. For octanol, we remark that since the duration of the first response is very long (> 60 s), the Ca^2+^-response of the first odor-application is not yet finished when the second odor application occurs (which likely explains why for the second application, the amplitude is higher than for the first one).

To determine if the odor-induced response in ORNs correlates with the odor-intensity, distinct groups of flies were exposed to either a 1 s or 5 s pulse of each odorant 5 successive times, with a 5 min-interval between each application. As reported in previous studies based on the use of various fluorescent probes, such as synapto-pHluorin [22,23] or G-CaMP [19,24] or GFP-Aequorin [18], a brief application (1 s) of an odorant increases the Ca^2+^-induced bioluminescence intensity in the ORNs. The amplitudes of evoked activities remain relatively constant during repetitive odor-stimulations for each of the three odors (Figure 2A,B,C). In contrast, a 5 s exposure causes a significant decrease in the amplitudes of Ca^2+^-responses during repetitive stimulations (Figure 2D,E,F) for the three odors. This decrease in the response is also visible in the duration (Figure 2J,K,L), which is odor-specific (spearmint = ~67 s; citronella = ~40 s; octanol = ~90 s, for the first application). Consequently, the total amount of emitted photons (ph), which depends on both the amplitude and the duration of the response, is also odor-specific (Figure 2G,H,I) (spearmint: ~12000 ph; citronella: ~6500 ph; octanol: ~17500 ph). Indeed, ORNs showed an attenuated response (total photons) to the second application, at about 50-70% of the magnitude of the first response to spearmint, citronella, and octanol. For the third application, the response was approximately 35-50% of the magnitude compared to the first exposure, for the fourth about 25-30%, a figure ultimately reduced to ~20-25% during the last stimulation, with the three odors following the same trend. This decrease could not be attributed to the GA exhaustion, since a longer odor application as long as 2 minutes generates a stronger (total photons) and continued response that remains for the entire duration of the odor application (though a decrease in the Ca^2+^-response due to the adaptation of the neurons can be observed), confirming that the availability of the bioluminescent probe is not a limiting factor (Figure 3A,B). In addition, to further support the fact that the amount of the GA probe is not a limiting factor, and consequently that the reduction of the Ca^2+^-response following successive odor applications reflects the adaptation process, we have used the Or22a-Gal4 line to express the GA in a specific glomerulus (DM2) [25]. Using the same protocol of odor-application (5s, 5X at 5 min-interval) (Additional file 2), we remark that the repetition of the odor stimulus induces a gradual reduction in the successive responses, revealing the adaptation. More importantly, as generally used for other Ca^2+^-probes [26], application of KCl at the end of the experiment (5 min after the fifth odor application), which depolarizes the neurons and induces a massive entry of calcium, induces the burning of all remaining probe. Additional file 2 clearly shows that a huge amount of GA probe was still available in these specific neurons (up to about 18000 photons). Therefore, the gradual decrease in the Ca^2+^-response cannot be due to an exhaustion of the GA, and thus represents an adaptation process. Moreover, as can be seen below, in some experimental conditions, a decrease in the amplitude of the response does not occur when different signaling pathways or neuronal networks that affect adaptation are blocked implying that GA is still capable of reacting to intracellular changes in Ca^2+ ^concentration. Finally, since adaptation has also been reported to depend on the frequency of the stimulus, increasing the frequency of the odor application (1 min-interval) generates an adaptation with slightly different kinetics, which seem to be odor-specific (compared to 5-min interval, it seems more pronounced for citronella, intermediate for spearmint and nearly identical for octanol) (Figure 3C). Together, these results demonstrate that ORNs rapidly adapt to odor stimuli, depending either on duration of the stimulus or frequency of its repetition. Although the odor responses to relatively short duration stimuli (< = 1 s) have been well documented [19,22,24,27,28], responses to longer applications, such as 5 s, have not been described in detail. As recently reported [18], the 5 s odor-induced response within the axon terminals of ORNs has two components: a first mechanism relying on VGCC and a delayed second mechanism that depends on the recruitment of intracellular Ca^2+^-stores. Thus, since 5 s odor application induces a strong and robust adaptation, we adopted these conditions (5 s, repeated at 5 min-interval, 5 successive times) to further characterize this long-lasting form of adaptation.

### Adaptation of Ca^2+^-response in axon terminals requires cholinergic synaptic transmission

**Figure 4:**
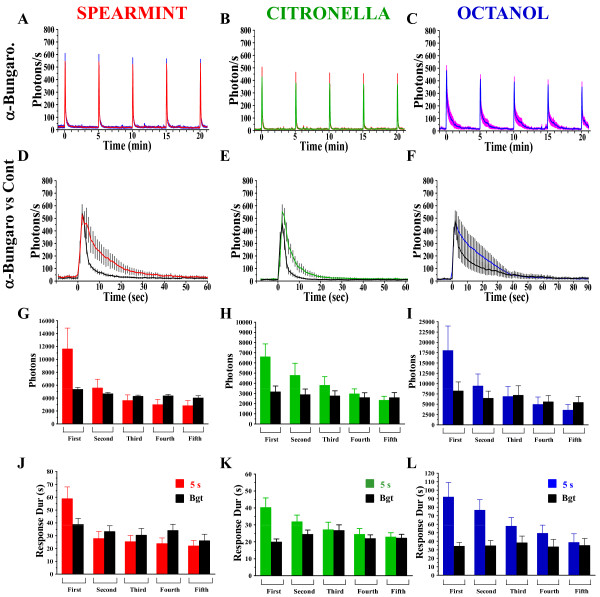
**The second component of the Ca^2+ ^-response depends on cholinergic synaptic transmission**. Mean (+/- SEM) amplitude of the overall responses (photons/s versus time) of the effect of α-bungarotoxin (Bgt) (A,B,C) on the Ca ^2+^-induced responses collected from the ROI, evoked by 5 application at 5-min interval, of a 5 s odor-duration of spearmint, citronella or octanol. (G-I) Mean of the total photons for each odor. (J-L) Mean of the duration(s) of the response. (D,E,F) Magnification view of the first odor application, showing the superposition of the Bgt-treated versus control flies (issued from Figure 2D,E,F respectively) for each odor. n = 5-9 flies for each condition. For the histograms (G-L): Values are means +/- SEM. Statistics: same tests as for Figure 2 (see Table 1 for statistical values).

First, odor-induced responses in ORNs generate spikes that propagate to the axon terminals, leading to the opening of the Voltage-Gated-Calcium-Channels (VGCC), responsible for transmitter (acetylcholine) release. Indeed, as recently reported [18], verapamil, a VGCC blocker [29] completely abolished the Ca^2+^-response in the antennal lobes for the three odors, indicating that presynaptic Ca^2+^-entry was necessary to trigger the Ca^2+^-response. Second, modulation of the ORNs-PNs synaptic transmission by local neuronal networks, either by feedback inhibition acting presynaptically (on ORNs) [27,28,30], or excitatory interactions acting post-synaptically (on PNs) [24,31] has been reported. To assess if the Ca^2+^-response monitored at the presynaptic terminal partly depends on neurotransmitter release and consequently on feedback control, we blocked synaptic transmission using α-bungarotoxin (Bgt), a specific blocker of the nicotinic acetylcholine receptors (nAchR) [29]. ORNs are indeed cholinergic neurons, while AchR (both muscarinic and nicotinic) have been reported in the antennal lobes [32,33]. However, exactly which neurons express which AchR still remain unclear. Application of Bgt reduced the first odor-induced response (total photons) for the three odors by about 60% (Figure 4G,H,I). More specifically, although the amplitude of the response remains consistent and does not decrease with odor repetition (Figure 4A,B,C), the overall response, represented by the total photons, was smaller (compared to control) and remains roughly constant for all applications (Figure 4G,H,I). Similarly, the duration of the response was much shorter as early as the first application, and remains constant for all successive applications (Figure 4J,K,L). Interestingly, these values roughly correspond to the first component (principally due to the VGCC: see Murmu et al., 2010 [18]), which is attributable to Ca^2+^ -entry into the terminals triggered by the spikes. Indeed, a higher magnification of the superposition of the odor-induced Ca^2+^ -response of the first odor-application following α-bungarotoxin drug-application over the control flies (Figure 4D,E,F) clearly shows that the second component described by Murmu et al., (2010) [18] is absent or severely reduced. These results suggest that in the presence of Bgt, the second component is not induced when nAChR are blocked; therefore it might result from an "excitatory/positive" feedback. Furthermore since the parameters for the subsequent responses (following repetitive stimuli) remain practically unchanged, it appears that this second component might be the main factor affected by the adaptation process.

### InsP_3_R and RyR are required to induce the second component, which amplifies the odor-induced response

**Figure 5:**
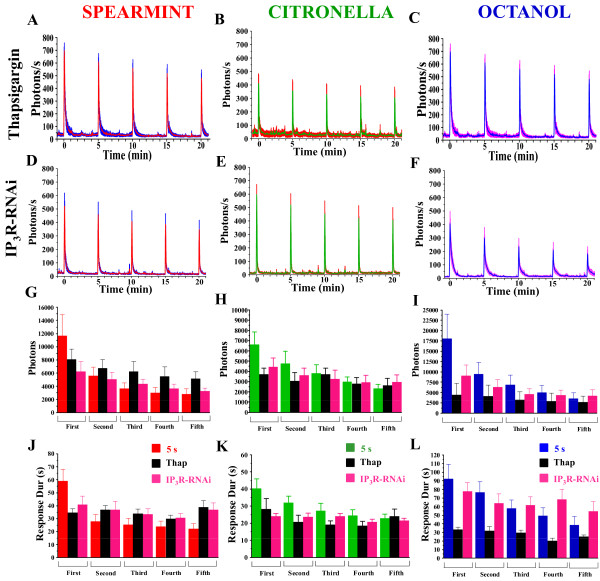
**Ca^2+^-transients in flies with pharmacologically blocked InsP_3_R or knocked-down InsP_3_R**. Response profiles of thapsigargin-treated flies (A-C), or Or83b,GA/InsP3R-RNAi flies (D-F) to 5 s application of spearmint, citronella, or octanol, applied 5 times at 5 min-interval. Although there is a tendency to decrease, the amplitude of evoked-activity during the repeated stimulations was not significantly different in the presence of thapsigargin or in InsP_3_R-RNAi-expressing flies for the 3 odors, except for spearmint and octanol in these latter flies. (G-I) Mean of the total photons for each condition. (J-L) Mean of the duration of the response (s). n = 5-9 flies for each condition. Values are means +/- SEM. Statistics: same tests as for Figure 2 (see Table 1 for statistical values).

Murmu et al. (2010) [18] have shown that interfering with InsP_3_R or RyR primarily decreases the second component of the Ca^2+ ^-response to odor application. Indeed these receptors are the two major channel/receptor complexes controlling the release of Ca^2+ ^from intracellular stores [34]. In *Drosophila*, mutation in InsP_3 _receptor gene (*itpr*) generates defects in olfactory adaptation [16]. To assess whether the InsP_3_R and/or RyR signaling pathways play a role in the adaptation of olfactory responses and in particular, serve to increase (or maintain) the presynaptic response, Ca^2+ ^-responses were analyzed using specific inhibitors or in flies with genetical defects in InsP_3_R or RyR signaling pathways in ORNs. Thapsigargin is an inhibitor of the endoplasmic reticulum (ER) Ca^2+ ^-ATPase which depletes intracellular Ca^2+^-stores [35]. Its application for 15 min (Figure 5A,B,C) decreases the total number of photons (Figure 5G,H,I) as well as the duration of the response (Figure 5J,K,L), mostly for the first and the second application of the odors, after which the decrease was less marked. Again, the peak amplitude of the response was not significantly different from that of control flies, nor did they show a significant decrease in response (adaptation) (Figure 5A,B,C). On the other hand, flies simultaneously expressing GA and an interfering RNA construct directed against the InsP_3_R (Or83b,GA/InsP_3_R-RNAi) show a reduction of light emission (total photons) (Figure 5G,H,I) during olfactory responses. They also present, to various extents, a decrease in the response duration (Figure 5J,K,L). However, the peak amplitude of the response was slightly decreased for spearmint and octanol, but not for citronella.

**Figure 6:**
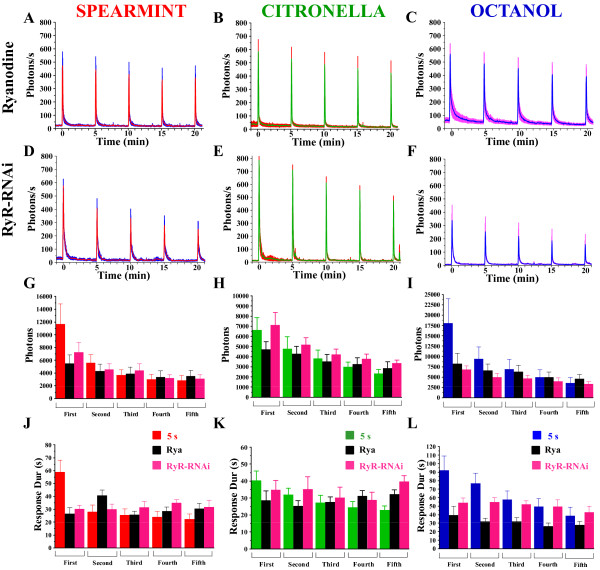
**Ca^2+^-transients in flies with pharmacologically blocked RyR or knocked-down RyR**. Response profiles of ryanodine-treated flies (A-C), or Or83b,GA/RyR-RNAi expressing flies (D-F) to 5 s application of spearmint, citronella, or octanol, applied 5 time, at 5 min-intervals. The decrease of the amplitude of the successive evoked transients was not significant in the presence of ryanodine for the 3 odors, contrary to that for the RyR-RNAi flies. (G-I) Mean of the total photons for each condition. (J-L) Mean of the duration of the response (s). n = 5-9 flies for each condition. Values are means +/- SEM. Statistics: same tests as for Figure 2 (see Table 1 for statistical values).

RyRs are expressed in the antennal lobes [36], although in which precise neuron remains unknown. Using a similar strategy, the contribution of the RyR to Ca^2+^-response was evaluated. At 100 μM and above, ryanodine is a blocker of the channel associated with the RyR [34]. Ryanodine incubation for 15-min decreases the response (total photons) (Figure 6G,H,I) and the duration of the response (Figure 6J,K,L) to spearmint and octanol but not to citronella, suggesting an odor-specific effect. Moreover, the amplitude does not decrease in response to the three odors (Figure 6A,B,C). Flies simultaneously expressing GA and a RNAi directed against the RyR (Or83b,GA/RyR-RNAi) show a decrease in response to olfactory stimuli for the three odors tested, both in total photons (Figure 6G,H,I), and response duration (Figure 6J,K,L). Moreover, though slightly different than ryanodine, compared to control flies, amplitude is affected, but they still adapt to repetitive stimuli (Figure 6D,E,F).

Overall, although some differences can be observed between the results obtained from the pharmacological versus a genetic approach and more specifically between ryanodine and the RyR-RNAi (although this last condition does not seems to completely block the adaptation, or solely in an odor specific manner) similar results were obtained with the two independent methods. These observations confirm that InsP_3_R as well as RyR, and consequently the Ca^2+ ^released from the intracellular Ca^2+^-stores contribute to the olfactory response, by allowing ORNs to increase and maintain their response according to increasing odor duration, and importantly, to adapt to a prolonged and/or repeated stimulus.

### Release of presynaptic Ca^2+^-stores within ORNs depends on GABAergic synaptic transmission in the antennal lobes

**Figure 7:**
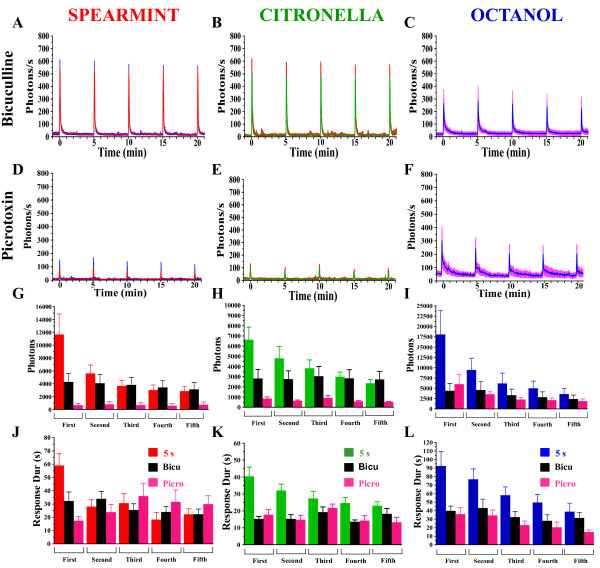
**Ca^2+^-transients in flies with pharmacologically blocked GABA_A _receptors**. Response profiles of bicuculline (A-C) or picrotoxin-treated flies (D-F) to 5 s application of spearmint, citronella, or octanol, applied 5 times at 5 min-intervals. The amplitudes of odor-evoked activity during the repeated stimulations remain constant for bicuculline for the 3 odors, as well as for picrotoxin, although they are strongly reduced for this last condition. (G-I) Mean of total photons for each condition. (J-L) Mean of the duration of the response (s). n = 5-9 flies for each condition. Values are means +/- SEM. Statistics: same tests as for Figure 2 (see Table 1 for statistical values).

Each glomerulus of the antennal lobes receives about 20 axons from the ORNs expressing the same receptor gene [37]. The axon terminals of the ORNs make synaptic contacts with dendrites of a few uniglomerular projections neurons (PNs), which propagate olfactory information to higher brain centers. Although the majority of PNs are cholinergic, some of them have been recently shown to be GABAergic [38,39]. The axon terminals of the ORNs also make contact with local interneurons (LNs), the majority of which are GABAergic [27,37]. Recently several other classes of LNs have been described, revealing an unexpected higher level of complexity [40]. Functionally, LNs can either be inhibitory [27,30,41] or excitatory [24,31,42,43] and modulate the processing of olfactory information, possibly by refining odor-tunings [28,30]. To study the possible contribution of the neuronal network, especially of the GABA (inhibitory or excitatory feedback control) on the ORN' axon terminals, we interfered with the GABAergic synaptic transmission both pharmacologically and genetically, given that majority of LNs are GABAergic. The *Drosophila *genome encodes three GABA_A _receptors subunits (RDL, LCCH3, GRD) [44], and two GABA_B _receptors [45]. GABA_A _receptors are pentameric and may exist as homomers or heteromers. Picrotoxin blocks RDL homomultimers, whereas RDL/LCCH3 heteromers are insensitive to picrotoxin but are blocked by bicuculline [46]. We monitored the response of individual flies to repeated odor-stimulation after applying the two GABA_A _receptor antagonists, bicuculline and picrotoxin (Figure 7). In the presence of bicuculline (250 μM), the amplitude of the first response to the three different odors was not affected as compared to controls (shown in Figure 2D,E,F). However, in contrast to the controls, the amplitude of the subsequent responses did not show any decrease (Figure 7A,B,C). Moreover, the total number of photons is significantly reduced (Figure 7G,H,I), while the duration of the response is shorter (Figure 7J,K,L), indicating that it roughly corresponds to the first (VGCC) component only. Picrotoxin (250 μM) strongly reduces the odor-induced response for the three odors tested (Figure 7). In contrast to bicuculline, the amplitude of the response is dramatically reduced after application of picrotoxin (Figure 7D,E,F). Consequently, the total number of photons is also dramatically reduced (Figure 7G,H,I), as well as the mean of the response duration (Figure 7J,K,L).

**Figure 8:**
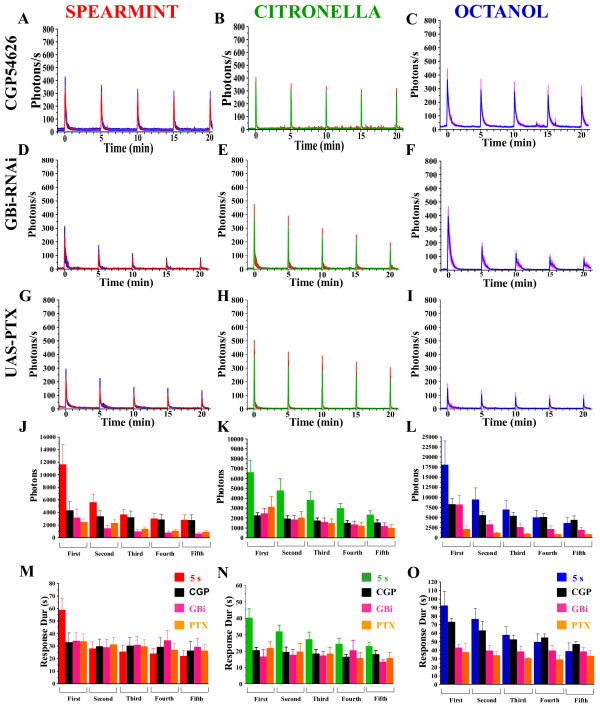
**Ca^2+^-transients in flies with disturbed GABA_B_R or G-proteins within the ORNs**. (A) Response profiles of CGP54626-treated flies (A-C), Or83b,GA/GABA_B_R2-RNAi (GBi) (D-F), or Or83b,GA/UAS-PTX flies (G-I) to 5 s application of spearmint, citronella, or octanol, applied 5 times at 5 min-intervals. The amplitudes of activity during the repeated stimulations do not decrease for CGP54626, for the 3 odors, while they are reduced to a large extent for GBi or UAS-PTX flies. (J-L) Mean of total photons for each condition. (M-O) Mean of duration of the response (s). n = 5-9 flies for each condition. Values are means +/-SEM. Statistics: same tests as for Figure 2 (see Table 1 for statistical values).

GABA_B _receptors are expressed on the axon terminals of ORNs [28]. To investigate the putative contribution of these receptors, we used CGP54626, a specific GABA_B_R antagonist. At 10 μM, CGP54626 modifies the amplitude of the odor-induced response to the first odorant application, and prevents a decrease in the amplitude of evoked Ca^2+^-activities during repetitive stimulations with the three odors tested (Figure 8A,B,C). However, it significantly decreases the total number of photons (Figure 8J,K,L), since the duration of the response is reduced (Figure 8M,N,O). Further, simultaneously expressing GA and a RNAi directed against the GABA_B_R2 (named GBi) in the ORNs (Or83b,GA/GBi-RNAi) impairs the odor-induced response (Figure 8). For the three odors, the amplitude of the response is reduced (compared to controls), while for spearmint and octanol (but not for citronella), it still adapts following repetitive odor application (Figure 8D,E,F) (even for octanol, the amplitude seems to adapt more rapidly). Moreover, similarly to CGP54626, the number of total photons is decreased (Figure 8J,K,L), as well as the duration of the response (Figure 8M,N,O). As in mammals, the GABA_B _receptor is a heterodimeric G protein [45,47]. Thus, to selectively abolish GABA_B _signaling in presynaptic ORNs, as previously described [30], we used the pertussin toxin (UAS-PTX), a selective inhibitor of several subtypes of G-proteins (Or83b,GA/UAS-PTX) [48]. Similarly to knocking-down the GABA_B_-receptor, disrupting G-protein signaling decreases the Ca^2+^-response. However, although a decrease is observed, it is not significant (Figure 8G,H,I) for the three odors. Again, although some differences could be observed between the different conditions used (bicuculline, picrotoxin, CGP54626, GBi-RNAi, UAS-PTX), which are also related to odor-specific differences, overall, relatively similar results obtained by independent approaches indicate that GABAergic synaptic transmission plays a crucial role in Ca^2+^-transients occurring in the ORN terminals, which might be involved in the control of Ca^2+^-release from intracellular Ca^2+^-stores.

### Blocking GABAergic signaling in ORNs yields functional defects in olfactory behavior

**Figure 9:**
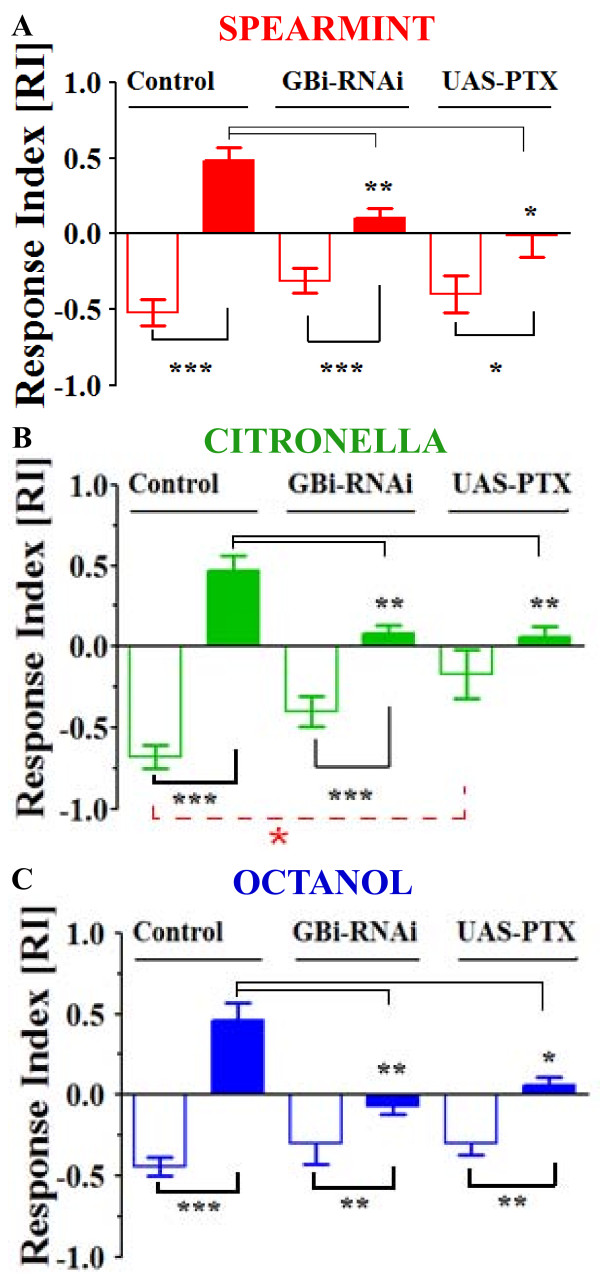
**Behavioral effects induced by disturbing the GABA_B_R or G-proteins within the ORNs**. (A,B,C) Response index of flies challenged with either spearmint, citronella or octanol in a T-maze without pre-exposure (hollow bars) or after pre-exposure (filled bars) for 5 min to the same odor. (Cont: Or83b,GA/CS; Or83b,GA/GBi-RNAi and Or83b,GA/UAS-PTX). For all groups, n = 10 batches of 10 flies/batch (100 flies per group). Values are means + SEM. Three types of comparisons have been performed: first, comparisons within the same genotype are made between those without and after pre-exposure; second, comparisons between control and the different genotypes are made for the "without pre-exposure condition" (all groups are non significant, except UAS-PTX for citronella); and third, comparisons between control and the different genotypes are made for the "after pre-exposure condition" (all groups are significantly different). (* P < 0,05; ** P < 0,001; *** P < 0,0001, Mann-Whitney U-test).

Olfactory acuity (level of the response to the first application of the stimulus) [16,49] has been dissociated from defects in adaptation, suggesting that the mechanism for cellular adaptation is independent of stimulus reception. We previously reported that disturbing the intracellular Ca^2+^-store release by knocking-down the InsP_3_R or the RyR within the ORNs yields olfactory behavior defects [18]. Here, similarly, we wondered if disrupting the GABAergic signaling specifically within the ORNs, which impedes adaptive Ca^2+^-response (Figure 8), could also yield specific defects at the behavioral level. In otherwords, what are the functional consequences for the fly of disrupting GABA signaling within the axon terminals of the ORNs? To address this question, we used the olfactory T-Maze test [18,49]. Flies were exposed to an odor and then tested with the same odor (at the same concentration) in the T-Maze (Figure 9). We recorded the behavioral response of sibling flies of three genotypes used for brain imaging: [Or83b,GA/CS] (control), [Or83b,GA/GBi-RNAi] and [Or83b,GA/UAS-PTX]. Pharmacologically manipulated flies cannot be used in this test. All appropriate controls (RNAi in heterozygous: GBi-RNAi/CS, and UAS-PTX/CS), were also tested, and did not show defects (data not shown). Without pre-exposure, all flies (control as well as those expressing GABA_B_R-RNAi or UAS-PTX) preferred the control arm (without odor), meaning that they were repelled (RI value between -0.3 to -0.7) by the odorant (Figure 9A,B,C), except for the UAS-PTX flies with citronella, for which the RI value is -0.20. To determine if odorant exposure affects the response to odors, sibling flies were pre-exposed to the odorants for 5 min. We found that after pre-exposure there was a significant change in the response of the control flies (Or83b,GA/CS): the preference toward the control-arm disappeared and the control flies then preferred the odor-arm for all three odors (p < 0.0001), mean RI value of ~0.5 (Figure 9). We interpret this "pre-exposure" effect as "adaptation". Interestingly, the inversion in the olfactory response, as a result of the pre-exposure, was significantly different in the Or83b,GA/GBi-RNAi and Or83b,GA/UAS-PTX flies. As shown (Figure 9), these flies had mean RI values around 0 indicating that they had become almost indifferent to the odor after pre-exposure. Thus, in contrast to the controls, which showed attraction to all three odorants after pre-exposure, these flies were unable to choose or could not discern between air and odor and hence were randomly distributed. Taken together, these results illustrate that olfactory behavior is severely impaired in flies lacking GABA_B_R or G-protein downstream signaling pathways, specifically in ORNs.

## Discussion

### Odor-induced Ca^2+^-responses within ORNs adapt to long-lasting or repetitive stimuli

This study provides evidence that the bioluminescent (GFP-aequorin) Ca^2+^-sensor is sensitive enough to monitor the Ca^2+^-response following various protocols (duration and repetition-frequency) of odor application. 1 s of odor induces a response which does not significantly decrease if repeated every 5 min, whereas a longer stimulus, such as 5 s, is sufficient to induce a decrease in response following repeated odor stimulations (adaptation) (Figure 2). Similarly, using a 5 s odor stimulation and increasing the frequency of repetition to 1-min intervals also induces, in an odor specific manner, a faster adaptation (Figure 3). We also demonstrate that prolonged odor application (up to 2 min) generates a sustained Ca^2+^-response within the ORN axon terminals (Figure 3), indicating that the ORNs are capable of responding as long as the odor is presented, of even longer (we can observe a short tail after stopping the odor). This work also indicates that the GFP-aequorin probe is not a limiting factor for the detection of the Ca^2+^-activity. These physiological results (reduction of the Ca^2+^-activity according to prolonged/sustained odor duration and/or odor repetition) are consistent with previous studies [2,3] which report that adaptation depends both on the duration of a stimulus and on the frequency of its repetition.

Different physiological approaches, based either on fluorescence brain imaging or electrophysiological techniques have previously reported odor-induced activity in different interconnected neurons in the antennal lobes of different invertebrate model organisms, including honeybees [50], locusts [51] and *Drosophila *[19,22-24,28,30,31,41] with the goal of deciphering the neural odor code. However, except for the study of Stopfer and Laurent (1999) [51] performed in locusts, which indirectly described a form of adaptation, long-lasting forms of adaptation within ORNs such as that described here has not been reported. This is likely due to the experimental design of these previous studies, which either generally took into account the odor-induced signal solely after the response was stabilized (generally after about 5 successive odor applications) [22], or used a shorter odor stimulation duration (< = 1 s), which as demonstrated here, is not sufficient to induce detectable and reliable adaptation. Additionally, others have relied on extracellular recordings of the sensillae of the antennae [27,30,31,41] which reflects the activity occurring in the cell-bodies of the ORNs. Here, monitoring the axon terminals of the ORNs, 5 s odor stimulations, repeated at 5-min intervals, induced a relatively long-lasting adaptation that resembles in term of kinetics, the long-lasting adaptation (LLA) reported by Zufall and Leinders-Zufall (1997) [9] in ORNs in salamanders. Indeed and interestingly, the recovery time (15 min for spearmint and octanol and 30 min for citronella) (Additional file 3) occurs over a similar time scale in salamander ORNs (which are different from the long-lasting olfactory adaptation described in *C. elegans *[52,53]). However, in contrast to LLA, which was reported in isolated ORNs, the adaptation described here seems to rely on different mechanisms, since it is sensitive to a "feedback control" provided by GABAergic synaptic transmission within the antennal lobes.

### Olfactory adaptation requires the recruitment of the intracellular Ca^2+^-stores

In *Drosophila*, mutants lacking InsP_3_R [16] are defective in olfactory adaptive behavior. In vertebrates, different forms of olfactory adaptation have also been reported in the ORNs [8,9,54]. First, we show in *Drosophila *that an adaptation mechanism occurs in axon terminal of the ORNs in the antennal lobes. Second, using two independent approaches, pharmacological and genetic, we show that odor-induced specific adaptation relies principally on InsP_3_R and RyR. When these two different receptors are blocked or knocked-down, although some difference (variability) can be observed between different conditions, overall the odor-induced Ca^2+^-response no longer adapts or is severely affected. More specifically, it seems that the lack of adaptation is due to the non-induction of the "second delayed and slow rising component" of the Ca^2+^-response, which is triggered in particular and specific conditions: when the duration of an odor stimulation is relatively long (here, in our experimental condition, 1 s does not induce it, while 5 s induces an important second component (see Murmu et al., 2010 [18] for a detailed description of the second component). Alternatively, the second component of the response is also induced and visible particularly on the first and to a lesser extent, on the second odor applications, especially when the odor is successively repeated. This second component gradually vanishes with sequential repetition. That is, we show that adaptation is not directly due to a decrease in the response, but rather indirectly to a defect in presynaptic Ca^2+^-increase, due to a lack of triggering release of intracellular Ca^2+^-stores, normally occurring in the first and successive responses following either a sufficiently strong (long stimulus) or repeated stimuli (Figure 2 and 3). These results suggest that one of the major intracellular mechanisms of adaptation depends on internal Ca^2+^-stores. In brief, we have blocked the intracellular mechanism that allows the cell to adapt to long lasting or repetitive stimuli. Interestingly, in mammals, in hippocampal CA3 pyramidal neurons, intracellular Ca^2+^-stores, which are controlled by InsP_3_R and/or RyR at the presynaptic terminal, have been previously implicated in neurotransmitter release as well as in synaptic plasticity [55,56].

### GABAergic synaptic transmission in the antennal lobes is required for adaptation

In vertebrates, neuronal plasticity related to odor representation occurs at the synapse between the ORNs and the second-order neurons in the olfactory bulb glomeruli, a region analogous to the invertebrate antennal lobes. At this synapse, signal transmission is modulated presynaptically by several mechanisms, a major one being via the metabotropic GABA_B _receptors. This suppresses presynaptic Ca^2+^-influx and subsequently transmitter release from the receptor neurons terminal [57,58]. At least two kinds of presynaptic inhibition (intra- and interglomerular) are mediated by GABA_B _receptors. Intraglomerular presynaptic inhibition seems to control input sensitivity [57,58], while interglomerular presynaptic inhibition seems to increase the contrast of sensory input [59] (although the two studies addressing this question *in-vivo *show contradictory results). In *Drosophila*, a similar mechanism seems to occur, as interglomerular presynaptic inhibition, mediated by both ionotropic and metabotropic receptors on the same axon terminal of the ORNs, mediate gain control mechanism, serving to adjust the gain of PN in response to ORN stimulation [30]. Yet another study has suggested that GABA_B _but not GABA_A _receptors are involved in presynaptic inhibition [28] yielding a contradiction. Here, by monitoring the Ca^2+^-release from the axon terminals of ORNs, in experimental conditions that generate a long-lasting form of adaptation, we have shown that GABAergic synaptic transmission plays a role in adaptation (Figures 7 and 8). Both ionotropic GABA_A_R antagonists, bicuculline and picrotoxin, block partially or completely the Ca^2+^-response, while, CGP54626, a metabotropic GABA_B_R antagonist, also blocks the adaptation, albeit not completely. It should be mentioned here that application of picrotoxin *per se *induces a strong transient Ca^2+^-release within the axon terminals of the ORNs, even without odor application (Additional file 4). This "transient release effect" likely disturbs the resting state of the neurons, which probably accounts for the important reduction observed in the amplitude of the odor-induced response. Nevertheless, these results suggest that both types of GABA receptors (A and B) are involved in adaptation. Moreover, as proposed by the study of Olsen and Wilson (2008) [30], it cannot be ruled-out that ORNs also express different subtypes of GABA_A_R (homo- and/or heteromultimers), since our results showed that picrotoxin and particularly bicuculline, two distinct inhibitors of GABA_A_R, block adaptation. Another possibility is that the effect of the two GABA_A_R antagonists results from the blockage of GABA_A_R on other neurons in the antennal lobes, as the LNs or certain PNs (which have not yet been demonstrated). Lastly and unfortunately, this pharmacological approach does not allow distinguishing by which precise neurons this GABAergic-dependent adaptation is mediated. With the goal of clarifying precisely in which neurons GABAergic transmission acts, we blocked the metabotropic GABA_B_R (GABA_B_R2-RNAi) or its signaling pathway (UAS-PTX) directly within ORNs. This yields defects in long-lasting adaptation for several conditions, seemingly in an odor specific manner (Figure 8). Therefore, although GABAergic effects have been described in ORNs of both *Drosophila *[28,30] and mammals [57,58,60], to support "feedback inhibition", we here report that in different experimental conditions such as a long odor duration (5 s) and/or repetition of the stimulus, it also participates in the adaptation process. Indeed, our results suggest that GABA signaling support a positive (excitatory) feedback control instead of an inhibitory feedback, as formerly reported by other studies [28,30]. Though these results seem to be contradictory, some explanations can be provided. First, as aforementioned, the experimental conditions are different: we used a relatively high odor concentration with relatively long odor duration (5 s). In addition, we recorded immediately from the first odor application and the successive one, while in the experimental protocol of certain studies, the odor is generally presented several times (priming) before the beginning of recording [22]. Consequently, it seems that these previous studies were performed on already adapted ORNs. This implies that the neuronal network in the antennal lobes was already stimulated, and therefore its dynamics was probably already modified, since as described here, an important effect occurs immediately after the first odor application. Moreover, GA allows monitoring, in continuity over a long time period, the intracellular level of calcium with high sensitivity to [Ca^2+^] (from ~ 10^-7 ^to 10^-3^). In addition, although we can't precisely assign which glomeruli are activated, this approach allows visualizing simultaneously the odor-induced Ca^2+^-activity from the entire antennal lobes (the overall depth). Therefore, we are monitoring the outcome of the overall response of the antennal lobes, instead of the response from single or a few glomeruli. Finally, in vertebrates it has been reported that in certain experimental conditions, GABA could be excitatory [for a review: [61], although this contradiction cannot yet be precisely explained. Furthermore, it seems that a given synapse can display inhibitory effects under one protocol and an excitatory effect with another. Notably, it has been reported that a short stimulation of GABA is inhibitory, while during a long stimulation, the GABA effect can switch from inhibitory to excitatory. Interestingly, this particular "switching effect" could potentially explain the current "contradictory" situation reported here: in our experimental conditions, in which we used a relatively long odor stimulus (5 s), GABA generates an excitatory effect, whereas in previous studies [28,30] based on short (<1 s) odor stimuli, GABA seems to be inhibitor. This difference in the duration of stimuli could perhaps account for such inverted or "switching effects".

### Blocking the GABAergic pathway within the ORNs disrupts olfactory behavior

To explore the behavioral and functional consequences of disturbing the GABAergic signaling pathway, we studied flies with a GABA_B_R2 (RNAi) ORN-specific genetic knockdown, as well those with a component of its signaling pathway, the G-protein, blocked by the pertussis toxin. Both groups of flies present strong behavioral deficits, as adaptation-disrupted flies are not able to discern between odors and air after 5-min of exposure to odor (Figure 9). Interestingly, control flies reverse their choice preferring odor after a 5-min pre-exposure (adaptation) suggesting that in these experimental conditions, the meaning of the odor changes in the fly's adapted state. These results are consistent with previous studies suggesting that adaptation could serve to extend the operating range of sensory systems over different stimulus intensities [1]. In other terms, adaptation modifies the sensitivity (threshold) to the odor, as previously reported in different organisms, such as *C. elegans *[6] and vertebrates [15] including humans [5]. This phenomenon is similar to that in other sensory modalities, as in visual system, where light adaptation in photoreceptors sets the gain, allowing vision at both high and low light levels [62]. As previously reported [18,63], odors could be repulsive (at high concentrations) or attractive (at low concentrations). In our experimental conditions in control flies the odors are repulsive. However, after 5-min of preexposure, the flies adapt to this odor concentration, and when tested at the same concentration odors are then likely only weakly perceived and therefore might correspond to an attractive "weak-odor concentration". In a former study in similar experimental conditions, we reported that the flies are attracted by each of these three odors for a weak odor concentration (see Figure S1 in [18]). Interestingly, reverse odor preference has already been reported in *C. elegans*, resulting from presynaptic changes involving a receptor-like guanylate cyclase (GCY-28) via the diacylglycerol/protein kinase C pathway [64]. Finally, the fact that without pre-exposure all groups of flies preferred the control arm and were repelled by the odorants indicates that the odor acuity of these flies is intact. In other words, odor-adaptation and not odor-acuity is affected in each of these groups of flies. These results strengthen the idea that odor perception and adaptation are indeed two distinct and separable processes.

## Conclusions

**Figure 10:**
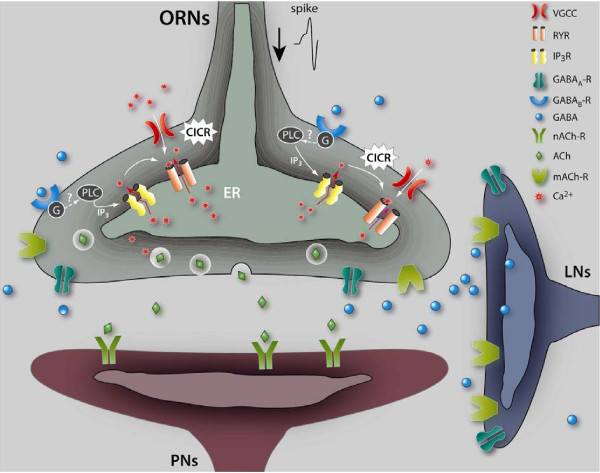
**Schematic model of synaptic interactions within the antennal lobes**. Spikes (action potentials) triggered by odors in ORNs propagate to the axon terminals, where they activate the VGCCs yielding to Ca^2+^-influx that serves as an intracellular signal to release the neurotransmitter (acetylcholine: Ach). This Ca^2+^-entry may also trigger Calcium-Induced Calcium-Release (CICR) through the ryanodine receptor (RyR) located in the endoplasmic reticulum (ER), amplifying the Ca^2+^-transient [34]. Subsequently, the released-Ach activates the AchR located on the post-synaptic neurons (projection neurons: PNs). Released-Ach might also activate (directly or indirectly) the GABAergic local interneurons (LNs) (in a manner that remains to be precisely determined), in-turn, releasing the GABA that may act on the presynaptic ORN terminals via the GABA receptors. Metabotropic GABA_B_R which has been reported on the axon terminals of the ORNs [28] may activate certain G-proteins (not yet precisely characterized in the ORNs), which for a relatively long odor application, such as 5 s (as in this study) might trigger the second component of the Ca^2+ ^-response by activating directly or indirectly the InsP_3_R (since blocking the G-proteins by the pertussis-toxin yields a similar effect to blocking directly the GABA_B_R). Therefore, we hypothesize that the GABA_B_R activated G-proteins might activate (directly or putatively indirectly through membrane channels) a phospholipase C (PLC) to catalyze the synthesis of diacylglycerol and InsP_3 _from PIP2 (phospho-inositol bis-phosphate). Interestingly, a former study has described a role for G_q_α, and phospholipase Cβ in insect olfactory transduction [66]. Activation of GABA_B_R in the ORN terminals might lead to InsP_3_-mediated Ca^2+ ^-release from the ER that could in turn also trigger CICR through RyR as a putative second step to amplify or maintain the Ca^2+ ^-transient. Some components of this pathway still remain to be investigated, such as the putative phospholipase C, as well as the different isoforms of G-proteins, and notably the G_q_α. In addition, our pharmacological results provide evidence that blocking the GABA_A_R disturbs the Ca^2+^-response within the ORNs to a large degree. However, which neurons in the antennal lobes express the ionotropic GABA_A_R has not yet been reported. Whether the GABA_A_R-effect occurs directly or indirectly on the ORNs remains to be investigated. Note that in this model the localization of the AchR (both muscarinic and nicotinic) and GABA_A_R are speculative, and remain to be determined.

We have demonstrated that the adaptation process occurring specifically in the axon terminals of the ORNs depends on intracellular Ca^2+^-stores, through InsP_3 _and ryanodine receptors. Moreover, we provide evidence that this Ca^2+^-release requires synaptic transmission, since it does not occur when the cholinergic receptors are blocked (α-bungarotoxin experiment). It also requires a feedback control through GAB A, since blocking GABA_B _signaling within ORNs prevents or strongly affects adaptation, suggesting that a local neuronal network mediated by GABAergic neurons is involved (for a more complete overview, see the schematic model of synaptic interactions within the antennal lobes, Figure 10). In complement to the brain imaging data, knocking-down the metabotropic GABA_B_R2, or its signaling pathway specifically in the ORNs, yields olfactory functional behavioral deficits. These results, combined with the results of blocking the InsP_3_R or RyR [18] suggest that a crucial olfactory integration process that can be ascribed to a form of neuronal plasticity and/or short-term memory occurs directly in the ORNs immediately after the first odor application or during a prolonged odor application. Thus, this effect could resemble the long-lasting form of odor adaptation described previously at the cellular and systems levels in vertebrates, including humans [5,65]. By extension, we hypothesize that in humans, the well-known "odor-specific transient functional anosmia" following a prolonged odor exposure, which results from an adaptation, may also rely on intracellular Ca^2+^-stores.

## Methods

### Flies

Flies were maintained on standard medium at RT (24°C). P[UAS-GFP-aequorin] (GA) transgenic flies [17] were used in conjunction with the P[GAL4]Or83b line, to target GA to the ORNs. P[GAL4]Or83b (Bloomington Stock Center) is expressed in a large population (approximately 80%) of sensory neurons [19]. Since both P[GAL4]Or83b and P[UAS-GA] are inserted on the 3rd chromosome, they were recombined onto the same chromosome, allowing further genetic crosses directly with the three different RNAi and UAS-PTX lines. Imaging experiments were performed on progeny of flies containing both the P[GAL4]Or83b driver and the P[UAS-GA] transgene (Or83b,GA/Canton-S) in transheterozygotes. We used specific RNAi (P[UAS-InsP_3_R-RNAi], P[UAS-RyR-RNAi] (R. Ueda, NIG, Japan) and P[UAS-GABA_B_R2-RNAi] (J. Wang, San Diego, USA), to knock-down the genes investigated specifically in the ORNs. We use the P[UAS-pertussin-toxin] (UAS-PTX) provided by G. Roman (Houston, USA), to inhibit some types of G-proteins. The P[GAL4]Or22a line was generated and provided by L. Vosshall (New York, USA).

### Brain preparation

Preparation of flies for live *in-vivo *brain imaging was performed as previously described [18]. In brief, a 4 day-old female fly was briefly cold (ice) anesthetized, inserted in a truncated 1 ml commercial pipette tip until the head protruded and was fixed in place with dental glue (Protemp III, ESPE™). The assembly was then placed in an acrylic block and secured with parafilm™. Ringer's solution [17] was deposited on the head, and a tiny window in the head capsule was cut out to expose the olfactory sensory neurons at the level of the antennal lobes. Care was taken not to damage the antennae. Then, the exposed brain was incubated in *Drosophila *Ringer's solution containing 5 μM native coelenterazine (Interchim, France) for 2 hours before experiments.

### *In-vivo *brain imaging

Odor-evoked bioluminescence signals in the ORNs were monitored with an electron multiplier CCD camera (EM-CCD, Andor, iXon, cooled to -80°C) fitted onto a microscope (Nikon, Eclipse-E800). The setup is housed inside a tight dark box (Sciences Wares, Inc., USA) to avoid any undesired (ambient) light contamination. We used a 20X dry-objective lens (N.A.: 0.75, Plan Apochromat, Nikon), giving a field of view of 400 × 400 μm (512 × 512 pixels). To improve signal to noise ratio, data were acquired with a 2 second integration time (0.5 Hz), and 2 × 2 binning was used (1 pixel = 1,56 × 1,56 μm). To acquire and store data, each detected photon was assigned x,y-coordinates and a time point. Response of individual flies to three different odors: spearmint, citronella, and octanol (3-Octanol) were recorded. The laboratory-made odor delivery apparatus consists of 5 identical channels, one of which is devoted to control air (without odor). From the air pump and a moistening bottle (containing 1 liter of water), each channel comprises a 50 ml flask with on either side a solenoid activated pinch-valve (Sirai S-104) isolating those not in use. All connecting tubes were made of silicone. Air flows continuously (500 ml/min) through the control channel except when a logic command issued by the imaging software switches the flow for the predetermined odor-duration time (1, 3, 5 s or 2-min) to one of the test (odor) channels. Test flasks contain 50 μl of undiluted pure odor (all from Sigma-Aldrich), deposited on a piece of filter paper. The air stream is delivered to the fly's antennae through a small glass tube placed a few millimeters away.

### Pharmacology

To interfere with calcium-induced calcium release (CICR), thapsigargin and ryanodine were used as previously described [18]. To investigate the roles of GABAergic synaptic transmission, GABA_A_-receptors antagonistic drugs such as bicuculline, picrotoxin and GABA_B_-receptors antagonistic drug as CGP54626 were used. Bicuculline (Fluka) was prepared as a 25 mM stock diluted further in *Drosophila *Ringers to 250 μM. Picrotoxin (Sigma) was prepared as 25 mM stock (50% distilled H2O: 50% ethanol) and dissolved in *Drosophila *Ringers up to 250 μM. CGP54626 (Tocris) was prepared as a 10 mM stock solution in 100% ethanol and diluted further in *Drosophila *Ringers at 10 μM final concentration. Prior to drug application, flies were stimulated once with odor to verify their responsiveness. The flies' brains were then incubated with either drug for 15-min. Afterward, the response of individual flies to repeated odor-stimulation was recorded.

### Behavioral Adaptation

Behavioral adaptation was measured by using an olfactory T-maze test [18]. Briefly, 10 flies, starved for 6 hours beforehand, were placed in the central chamber, in an upper position. The central chamber was set to the bottom position, from which flies, given a maximum of 15 s, could choose between the two side arms (chambers). For testing adaptation, the flies were pre-exposed to the odorant for 5 min in a top chamber and then moved down, via the central chamber, to choose between the control-air and the odor-containing airstreams. In both cases, the total number of flies in each side chamber was counted. The response index [RI] was calculated by subtracting the number of flies in an odorant-containing channel from the number of flies in the control arm and dividing by the total number of flies. The RI value ranged between 1 to -1. If all flies were repelled by an odorant, the RI would equal -1.0, whereas, if all were attracted, the RI would equal 1.0. RI equals 0 if the flies were indifferent to the odorant (randomly distributed).

### Quantitative and Statistical Analysis

We used the Photon Viewer (1.0) software (Sciences Wares, Inc., USA) written in LabView 7.1 (National Instruments) to analyze imaging data. Odor-evoked bioluminescence signals are presented as photons/s (within the ROI). Image recordings were obtained from 5-9 flies for each genotype. For the olfactory T-maze, 10 groups of 10 flies (100 flies) for each genotype were analyzed and averaged. All statistics were done using the Statistica (7.1) software (StatSoft, Inc.). A one-way analysis of variance was used to test adaptation (reduction in the amplitude of evoked Ca^2+^-responses during the repetitive odor-stimulation) in the control flies (Or83b,GA/CS) as well in all groups of flies. We used a two-way analysis of variance using treatment as the first factor and time as the second factor to determine differences in adaptation between the control (Or83b,GA/CS) and the experimental groups (Or83b,GA/InsP_3_R-RNAi, Or83b,GA/RyR-RNAi, Or83b,GA/GBi-RNAi, Or83b,GA/UAS-PTX and pharmacologically-treated flies). The Mann-Whitney test was used to test for significant differences in the olfactory responses of control and experimental flies in the T-maze choice test.

## Authors' contributions

The author(s) have made the following declarations about their contributions: Conceived and designed the experiments: MSM, JRM. Performed the experiments: MSM, ER. Analyzed the data: MSM, ER, JS, JRM. Contributed reagents/materials/analysis tools: MSM, JS, JRM. Wrote the paper: MSM, JS, JRM. All authors read and approved the final manuscript.

**Table 1:** Statistical significance of different tested conditions

Conditions	Spearmint	Citronella	Octanol
**One-Way ANOVA**			

**Control 5 sec**	A (***), S (**), D (**)	A (***), S (*), D (*)	A (***),S (*), D (*)

**Control 1 sec**	A (ns), S (ns), D (ns)	A (ns), S (ns), D (ns)	A (ns), S (ns), D (ns)

α**-Bungaro**.	A (ns), S (ns), D (ns)	A (ns), S (ns), D (ns)	A (ns), S (ns), D (ns)

**Thapsigargin**	A (ns), S(ns), D (ns)	A (ns), S (ns), D (ns)	A (ns), S (ns), D (ns)

**InsP**_**3**_**R-RNAi**	A (*), S (ns), D (ns)	A (ns), S (ns), D (ns)	A (*), S (ns), D (ns)

**Ryanodine**	A (ns), S(ns), D (ns)	A (ns), S (*), D (ns)	A (ns), S (ns), D (ns)

**RyR-RNAi**	A (**), S (*), D (ns)	A (***), S (*), D (ns)	A (*), S (ns), D (ns)

**Bicuculline**	A (ns), S (ns), D (ns)	A (ns), S (ns), D (ns)	A (ns), S (ns), D (ns)

**Picrotoxin**	A (ns), S(ns), D (ns)	A (ns), S (ns), D (ns)	A (ns), S (ns), D (ns)

**CGP54626**	A (ns), S (ns), D (ns)	A (ns), S (ns), D (ns)	A (ns), S (ns), D (ns)

**GBi-RNAi**	A(***), S (ns), D (ns)	A (ns), S (ns), D (ns)	A (***), S (*), D (ns)

**UAS-PTX**	A (***), S (ns), D (ns)	A (ns), S (ns), D (ns)	A (***), S (ns), D (ns)

			

**Two-Way ANOVA**			

**Cont: 5 s vs 1 s**	A (***), S (**), D (***)	A (***), S (**), D (***)	A (***), S (*), D (*)

**Cont vs **α**-Bunga**.	A (***), S (*), D (*)	A (***), S (*), D (*)	A (*), S (*), D (*)

**Cont vs Thapsigargin**	A (***), S (*), D (*)	A (***), S (*), D (*)	A (***), S (**), D (*)

**Cont vs InsP**_**3**_**R-RNAi**	A (***), S (*), D (*)	A (***), S (*), D (*)	A (***), S (*), D (*)

**Cont vs Ryanodine**	A (***), S (*), D (**)	A (***), S (*), D (*)	A (***), S (*), D (**)

**Cont vs RyR-RNAi**	A (***), S (**), D (*)	A (***), S (**), D (*)	A (***), S (*), D (*)

**Cont vs Bicuculline**	A (***), S (*), D (*)	A (***), S (*), D (*)	A (***), S (*), D (*)

**Cont vs Picrotoxin**	A (***), S (***), D (*)	A (***), S (**), D (**)	A (***), S (*), D (***)

**Cont vs CGP54626**	A (***), S (*), D (*)	A (***), S (***), D (***)	A (**), S (*), D (***)

**Cont vs GBi-RNAi**	A (***), S (*), D (*)	A (***), S (**), D (**)	A (***), S (*), D (*)

**Cont vs UAS-PTX**	A (***), S (*), D (*)	A (***), S (***), D (***)	A (***), S (*), D (***)

For the three quantified parameters (Amplitude, Sum and Duration), to check if the responses decrease (adapt), One-Way ANOVA have been performed. For the total photons and duration, Two-Way ANOVA using treatment as the first factor and time as the second factor to determine differences in adaptation between the control (Or83b,GA/CS: 5 s odor application) and the experimental groups have been performed. (* P < 0,05; ** P < 0,001; *** P < 0,0001). A = Amplitude, S = Sum of total photons, D = Duration of the response.

## Supplementary Material

Additional file 1 ***In-vivo *bioluminescence imaging of Ca^2+^-responses elicited at the level of the antennal lobes in the olfactory sensory neurons (ORNs), of a living OR83b,GA/CS fly during 5 s of 5 successive applications, at 5 min-intervals of each of the three odors; spearmint, citronella, octanol, respectively**. We note that the localization of the odor-evoked response is different for each odor, suggesting a specific glomerular activity pattern. Each frame represents 20 s of light accumulation and is shifted by 7 s (25 frames/s). The movie is seen 175 times faster. The light emission is coded in pseudocolors (0-6 photons/pixel) (QuickTime: 9,78 Ko). Click here for file

Additional file 2 **KCl response of specific ORNs targeted by OR22a-GAL4 following a standard odor-induced adaptation protocol**. Mean (+/- SEM) of the total amount of emitted photons of the Ca^2+^-induced response (within the ROI) evoked by the standard adaptation protocol (5 s of citronella, 5 successive applications, at 5-min intervals). KCl (70 mM) is applied 5 min after the last odor application. Since KCl depolarizes the neurons and induces a massive entry of calcium, it burns all remaining GA probe. We remark that KCl response goes up to about 18000 photons (about 18X higher than the fifth odor application), demonstrating that large amounts of GA probe were still available after the fifth odor application, implying that the gradual decrease of the response following the 5 successive odor applications is due to an adaptation process, and conversely, that GA is not a limiting factor. Click here for file

Additional file 3 **Recovery adaptation time of each odor**. Mean (+/- SEM) amplitude of the response (photons/s) of different flies, versus time, of the Ca^2+ ^-induced response (within the ROI) evoked by a 5 s application of spearmint (A), citronella (B) or octanol (C). A period of at least 15-min (recovery time period) is required between two applications for spearmint and octanol to obtain a response of a similar amplitude compared to the first odor-application, while it takes at least 30 min for citronella, suggesting that recovery time from olfactory adaptation is odor-dependent. Click here for file

Additional file 4 **Application of picrotoxin induces a transient Ca^2+^-release**. Mean (+/- SEM) amplitude of the response (photons/s) versus time of the Ca^2+ ^-induced response (within the ROI) evoked by picrotoxin application (250 μM). This result clearly demonstrates that picrotoxin application induces, *per se*, a release of Ca^2+^, which extends for about 10 to 15 min. Note that the amount of released calcium is much lower than the GFP-aequorin capacity to respond to the stimulus. Indeed, the mean of the sum of total photons emitted after picrotoxin application is 11159 for the overall antennal lobes (emitted from all OR83b targeted neurons), while, as an example, GA is able to emit more than 35000 photons just for the few spearmint excited neurons (as compared to Figure 3B1). Finally, remark also that the SEM is larger and more variable after picrotoxin application than before (even difficult to observe at this magnification). (Mean = red line, SEM = blue). Click here for file
