# Supplementary figures and images for: Calcium-stores mediate adaptation in axon terminals of Olfactory Receptor Neurons in Drosophila
Source: BMC Neurosci. 2011 Oct 24;12:105. doi: 10.1186/1471-2202-12-105 (PMC3226658; doi:10.1186/1471-2202-12-105)

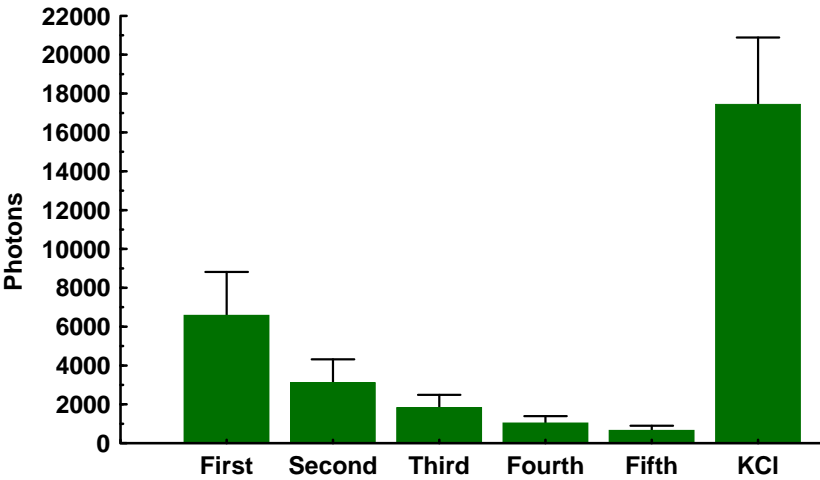

Supplement: Additional file 2 — KCl response of specific ORNs targeted by OR22a-GAL4 following a standard odor-induced adaptation protocol. Mean (+/- SEM) of the total amount of emitted photons of the Ca2+-induced response (within the ROI) evoked by the standard adaptation protocol (5 s of citronella, 5 successive applications, at 5-min intervals). KCl (70 mM) is applied 5 min after the last odor application. Since KCl depolarizes the neurons and induces a massive entry of calcium, it burns all remaining GA probe. We remark that KCl response goes up to about 18000 photons (about 18X higher than the fifth odor application), demonstrating that large amounts of GA probe were still available after the fifth odor application, implying that the gradual decrease of the response following the 5 successive odor applications is due to an adaptation process, and conversely, that GA is not a limiting factor. [file 1471-2202-12-105-S2.PDF]

# SPEARMINT

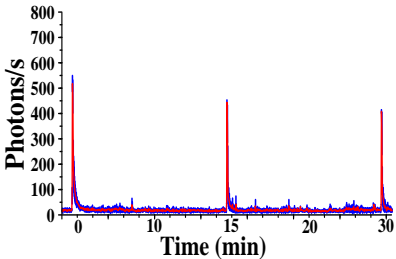

# CITRONELLA

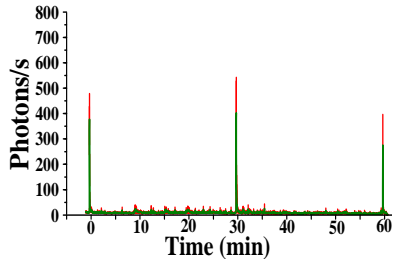

# OCTANOL

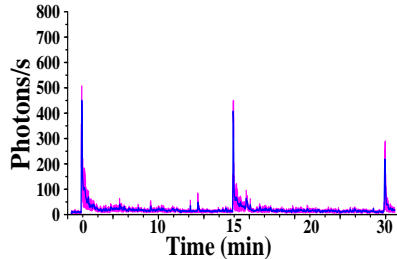

Supplement: Additional file 3 — Recovery adaptation time of each odor. Mean (+/- SEM) amplitude of the response (photons/s) of different flies, versus time, of the Ca2+ -induced response (within the ROI) evoked by a 5 s application of spearmint (A), citronella (B) or octanol (C). A period of at least 15-min (recovery time period) is required between two applications for spearmint and octanol to obtain a response of a similar amplitude compared to the first odor-application, while it takes at least 30 min for citronella, suggesting that recovery time from olfactory adaptation is odor-dependent. [file 1471-2202-12-105-S3.PDF]

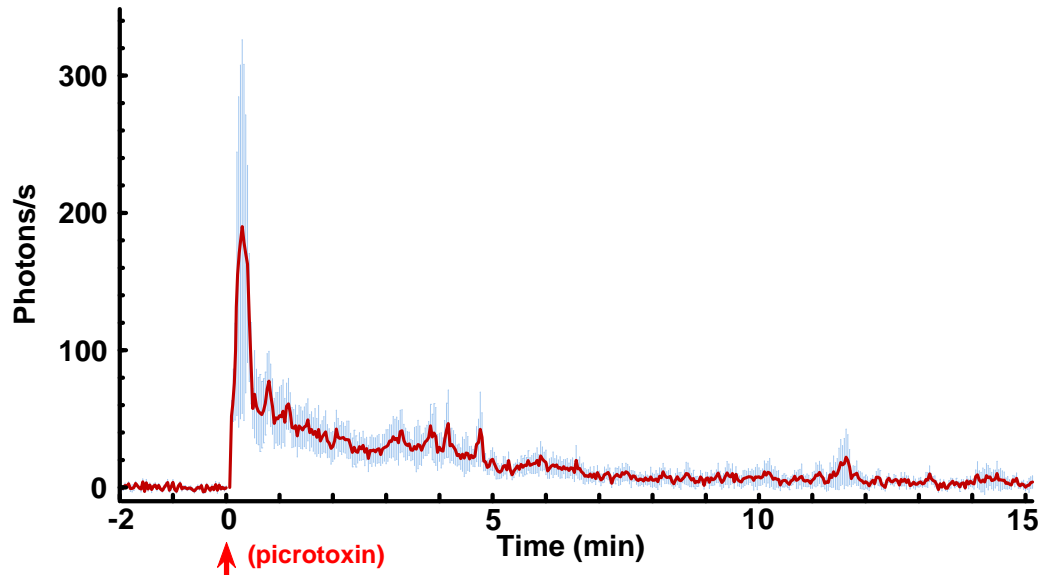

Supplement: Additional file 4 — Application of picrotoxin induces a transient Ca2+-release. Mean (+/- SEM) amplitude of the response (photons/s) versus time of the Ca2+ -induced response (within the ROI) evoked by picrotoxin application (250 μM). This result clearly demonstrates that picrotoxin application induces, per se, a release of Ca2+, which extends for about 10 to 15 min. Note that the amount of released calcium is much lower than the GFP-aequorin capacity to respond to the stimulus. Indeed, the mean of the sum of total photons emitted after picrotoxin application is 11159 for the overall antennal lobes (emitted from all OR83b targeted neurons), while, as an example, GA is able to emit more than 35000 photons just for the few spearmint excited neurons (as compared to Figure 3B1). Finally, remark also that the SEM is larger and more variable after picrotoxin application than before (even difficult to observe at this magnification). (Mean = red line, SEM = blue). [file 1471-2202-12-105-S4.PDF]
